# E2112—Does a negative phase III trial of endocrine therapy plus histone deacetylase inhibitor in hormone receptor‐positive advanced breast cancer represent a death knell?

**DOI:** 10.1111/1759-7714.14399

**Published:** 2022-03-29

**Authors:** Liyi Zhang

**Affiliations:** ^1^ Sun Yat‐sen University Cancer Center State Key Laboratory of Oncology in South China; Collaborative Innovation Center for Cancer Medicine Guangzhou China

According to the Global cancer statistics, breast cancer tops the list of the most common cancers in 2020.^1^ With the largest population in the world, China plays an important role in the effort of breast cancer burden and control.^2^, ^3^, ^4^ Each subtype of breast cancer has distinct molecular characteristics. At present, classification of breast cancer patients based on the molecular characteristics to determine prognosis and recommend treatment options has become the gold standard practice. Hormone receptor‐positive (HR^+^) and human epidermal growth factor receptor 2‐negative (HER2^−^) breast cancer represents the most common subset in late‐stage settings. Endocrine therapy is recognized as the most effective treatment strategy for HR^+^HER2^−^ breast cancer. However, drug resistance is a major obstacle that limits the success of endocrine therapy in breast cancer.^5^ Recent studies have demonstrated that combining endocrine therapy with drugs that target resistance mechanisms can delay the development of endocrine therapeutic resistance.^6^ Therefore, it is necessary to develop more effective combination treatment strategies to combat resistance to endocrine therapy.

Epigenetic modifications that can alter gene expression are one of the main causes of breast cancer progression and endocrine therapy resistance.^7^ Previous studies have shown that histone deacetylase (HDAC) inhibitors, an epigenetic modifier, can reverse endocrine therapy resistance.^8^ Entinostat is a selective class I HDAC inhibitor undergoing clinical trials for the treatment of multiple solid tumors. It can downregulate the estrogen‐independent growth factor signaling pathway and promote the normalization of estrogen receptor levels by inducing protein lysine acetylation.^9^, ^10^ Additionally, entinostat has been proven to successfully reverse letrozole resistance in mouse models.^8^ Based on these findings, the randomized phase II clinical study, ENCORE301, evaluated the safety and efficacy of steroidal aromatase inhibitor (AI) plus entinostat in patients with advanced HR^+^ and HER2^−^ breast cancer.^11^ This study reported that the addition of entinostat to the steroidal AI could improve both overall survival and progression‐free survival, and the level of protein lysine acetylation in peripheral blood mononuclear cells was associated with the prolonged progression‐free survival in the entinostat group. Exemestane is a third‐generation steroidal AI that has demonstrated efficacy in the treatment of postmenopausal patients with advanced breast cancer. However, the safety and efficacy of adding entinostat to exemestane in the treatment of advanced HR^+^ and HER2^−^ breast cancer remains unknown. In a study recently published in the *Journal of Clinical Oncology*, titled “E2112: Randomized Phase III Trial of Endocrine Therapy Plus Entinostat or Placebo in Hormone Receptor‐Positive Advanced Breast Cancer. A Trial of the ECOG‐ACRIN Cancer Research Group”, Connolly et al.^12^ conducted a multicenter, randomized, phase III study (E2112) to evaluate these issues.

In this study, 608 patients with advanced HR^+^ and HER2^−^ breast cancer were enrolled and randomly assigned to exemestane plus entinostat arm (*n* = 305) and exemestane plus placebo arm (*n* = 303) between March 2014 and October 2018. They ranged in age from 29 to 91 years (median = 63 years). Among these patients, 511 (84%) patients had disease progression after receiving nonsteroidal AI treatment in a metastatic setting and 365 (60%) had visceral disease. In addition, 60% of patients had received chemotherapy, 30% had received fulvestrant, and 35% had received cyclin‐dependent kinase inhibitor before enrollment. In the exemestane plus entinostat arm, the most common grade 3 and 4 adverse events were neutropenia (20%), hypophosphatemia (14%), anemia (8%), leukopenia (6%), fatigue (4%), diarrhea (4%), and thrombocytopenia (3%). There was no significant difference between the exemestane plus entinostat arm and the exemestane plus placebo arm in either progression‐free survival or overall survival (median progression‐free survival: 3.3 vs. 3.1 months; median overall survival: 23.4 vs. 21.7 months; hazard ratio = 0.99; 95% confidence interval = 0.82–1.21; *p* = 0.94). The objective response rate in the exemestane plus entinostat arm and the exemestane plus placebo arm was 5.8% and 5.6%, respectively. Target inhibition was confirmed by pharmacodynamic analysis in patients who received entinostat.

The E2112 phase III trial is a randomized, double‐blind, placebo‐controlled trial to test whether the addition of entinostat to exemestane improves the outcomes of patients with AI‐resistant, HR^+^, and HER2^−^ advanced breast cancer. Unfortunately, it did not meet either of the coprimary endpoints, suggesting that the addition of entinostat to exemestane did not significantly improve patient outcomes. Although both previous preclinical studies in mouse models with AI‐resistant breast cancer^8^ and randomized phase II clinical trial (ENCORE301)^11^ obtained positive data, this does not mean that similar results can be achieved in a phase III clinical trial. A well designed phase III trial is the best way to find a novel treatment standard. The study design of the E2112 trial is similar to that of the ENCORE301 trial. Their inclusion criteria included prior resistance to AI, postmenopausal status, allowance of prior chemotherapy (*n* ≤ 1) for metastatic disease, and no prior fulvestrant treatment. In both studies, the upper limit of enrolment for patients with unmeasurable disease was 20%. However, the E2112 trial made some adjustments to the inclusion criteria in the initial phases and permitted enrollment of premenopausal patients with previous use of fulvestrant or concurrent ovarian suppression. Ultimately, the E2112 trial enrolled 8% of premenopausal patients, 30% of patients previously treated with fulvestrant, and 35% of patients who had previously received cyclin dependent kinases inhibitors. These changes may affect the results to a certain extent.

The ACE trial, a randomized, placebo‐controlled, phase III trial, reported progression‐free survival advantages in contrast to the E2112 trial,^13^ and China Food and Drug Administration approved the combination of exemestane and tucidinostat (a HDAC inhibitor) in breast cancer. In the ACE trial, postmenopausal patients who experienced disease recurrence/progression after at least one endocrine therapy were randomly assigned (ratio = 2:1; *n* = 365) to exemestane plus tucidinostat or placebo. Results of this trial showed that exemestane plus tucidinostat extended progression‐free survival by 3.6 months.^13^ The E2112 and ACE trials differed significantly in the study populations (the ACE trial enrolled patients in North America and the E2112 trial enrolled patients in China), thus, genetic differences between the two study groups may have affected the individual response to the HDAC inhibitor. In addition, patients enrolled in the ACE trial were younger than those enrolled in the E2112 trial (median age = 55 years in the ACE trial; median age = 63 years in E2112 trial) and the patients of the E2112 trial in the advanced‐disease setting were more likely to have received prior endocrine therapy (84% in E2112 trial; 50% in ACE trial). The E2112 and ACE trials also differ widely in results. The frequency of grade 3/4 adverse events observed in the study arm of the E2112 trial was much lower than that observed in the ACE trial (50% in the E2112 trial; 75% in the ACE trial), suggesting that entinostat and tucidinostat are different in the degree of HDAC inhibition and off‐target effects.^14^, ^15^ Although the data of the E2112 trial indicated that endocrine therapy plus HDAC inhibitor did not improve progression‐free survival, progression‐free survival improved by 3.6 months in the ACE trial, suggesting that whether the use of HDAC inhibitor entinostat in the E2112 trial has real clinical significance still needs further verification and better predictors are needed to identify those patients who could benefit from endocrine therapy plus HDAC inhibitor.

One of the strengths of the E2112 trial is its robust, randomized, double‐blind, placebo‐controlled design. Moreover, other strengths include a strong supportive preclinical/clinical rationale, coprimary objectives of progression‐free and overall survival, and a large cohort of patients recruited via the National Cancer Institute National Clinical Trials Network. Although the E2112 trial did not achieve positive results, the data it collected provide a rich resource for further study of the prognostic/predictive factors in endocrine‐resistant breast cancer.

Overall, the E2112 trial suggests that exemestane plus entinostat fails to prolong survival in advanced endocrine‐resistant breast cancer patients. However, whether HDAC inhibitors can play an effective therapeutic role in biomarker selected breast cancer populations still needs to be further investigated.

## CONFLICT OF INTEREST

The author declares no competing interests.
